# Abnormal Anatomical Variations of Extra-Hepatic Biliary Tract, and Their Relation to Biliary Tract Injuries and Stones Formation

**DOI:** 10.14740/gr596e

**Published:** 2014-03-14

**Authors:** Meiaad F. Khayat, Munaser S. Al-Amoodi, Saleh M. Aldaqal, Abdulrahman Sibiany

**Affiliations:** a Department of Anatomy, Faculty of Medicine (Rabigh Branch), King Abdulaziz University, Jeddah, Saudi Arabia; b Department of Surgery, Faculty of Medicine, King Abdulaziz University, Jeddah, Saudi Arabia

**Keywords:** Extra-hepatic biliary tract, Anatomical variations, Biliary tract injury, Biliary stones

## Abstract

**Background:**

To determine the most common abnormal anatomical variations of extra-hepatic biliary tract (EHBT), and their relation to biliary tract injuries and stones formation.

**Methods:**

This is a retrospective review of 120 patients, who underwent endoscopic retrograde cholangiopancreaticography (ERCP) and/or magnetic resonance cholangiopancreaticography (MRCP), between July 2011 and June 2013. The patients’ ERCP and MRCP images were reviewed and evaluated for the anatomy of EHBT; the medical records were reviewed for demographic data, biliary tracts injuries and stones formation.

**Results:**

Out of 120 patients, 50 were males (41.7%) and 70 were females (58.3%). The mean age was 54 years old (range 20 - 88). Abnormal anatomy was reported in 30% (n = 36). Short cystic duct (CD) was found in 20% (n = 24), left CD insertion in 5% (n = 6), CD inserted into the right hepatic duct (RHD) in 1.7% (n = 2), duct of Luschka in 3.33% (n = 4) and accessory hepatic duct in also 3.33% (n = 4). Biliary tract injuries were reported in 15% (n = 18) and stones in 71.7% (n = 86). Biliary tract injuries were higher in abnormal anatomy (P = 0.04), but there was no relation between abnormal anatomy and stones formation.

**Conclusion:**

Abnormal anatomy of EHBT was found to be 30%. The most common abnormality is short CD followed by left CD insertion. Surgeons should be aware of these common abnormalities in our patients, hence avoiding injuries to the biliary tract during surgery. The abnormal anatomy was associated with high incidence of biliary tract injury but has no relation to biliary stone formation.

## Introduction

Extra-hepatic biliary tract (EHBT) is one of the most common sites of surgical procedures. It is important for the surgeon to be aware of the EHBT anatomy and be able to identify its possible abnormal anatomical variations, as the presence of these variations may increase the likelihood of biliary tract injuries during surgery [1, 2].

Incidence of EHBT anatomical variations was reported to be as much as 47% [1]. These variations include: accessory hepatic ducts; aberrant ducts communicating liver directly to the gall bladder (accessory cysticohepatic ducts) or ducts of Luschka; low insertion of the cystic duct (CD), insertion of the cystic duct into right or left hepatic duct (RHD or LHD); CD insertion in the left side of the common hepatic duct (CHD) or left CD insertion [2]; short CD, long CD and double CD [3, 4].

The aim of this study is to determine the most common abnormal anatomical variations of EHBT, and their relation to biliary tract injuries and stones formation, making surgeons aware of the common abnormalities, hence avoiding biliary tract injury during surgery. According to our knowledge, this is the first study in our region discussing the anatomical variations of EHBT.

## Methods

A retrospective study of all patients, who underwent endoscopic retrograde cholangiopancreaticography (ERCP) and/or magnetic resonance cholangiopancreaticography (MRCP) for different reasons between July 2011 and June 2013 (a total of 120, both males and females) at King Abdulaziz University Hospital (KAUH), was undertaken. Our hospital is the only university teaching hospital and one of four tertiary hospitals in the western region of Saudi Arabia, with capacity of 754 beds.

Patients’ medical records, ERCP and MRCP images were reviewed and evaluated after we obtained approval from the local ethics committee. Data sheet was developed and divided to five sections. The first section included the patient’s age, gender and the dates when the ERCP and MRCP images were taken. The second section evaluated the lengths of RHD, LHD, CHD, CD, common bile duct (CBD) and pancreatic duct (PD). Evaluation of cysticohepatic angle and hepatic bifurcation angle was also considered. The third section described the anatomical variations of the EHBT and PD, and determined the abnormalities including abnormal insertions and presence of accessory ducts. The fourth section included the history of biliary stones, cholecystectomy and bile duct injuries. The last section was designed for a printed image of ERCP and MRCP.

The data were entered and analyzed using the statistical package for social sciences (SPSS Inc., Chicago, IL, USA), version 20.00. The quantitative data were presented in the form of mean, standard deviation and range. Chi-square test was performed to compare qualitative data: anatomy (normal/abnormal) with bile duct injuries and anatomy with biliary stones formation. Independent t-test was used to compare CBD length between males and females. Pearson’s correlation test was performed to correlate CBD length with age. We considered statistical significance when P value < 0.05 and confidential interval of 95%.

## Results

A total of 120 patients underwent ERCP and/or MRCP. Fifty patients were males (41.7%) and the other 70 were females (58.3%). The mean age was 53.85 ± 18.66 years (range 20 - 88). The mean length of the CHD was 2.19 ± 1.01 cm (range 1 - 5.1 cm), CBD 7.21 ± 2.19 cm (range 3.19 - 12.89 cm) and CD 2.86 ± 1.68 cm (range 0.40 - 8.38 cm). Other measurements of EHBT and PD are shown in Table 1.

**Table 1:** Measurements of EHBT and PD

	Mean length and standard deviation (cm)	Range (cm)
Right hepatic duct (RHD)	1.77 ± 0.79	0.51 - 4.72
Male	2.08 ± 0.94	
Female	1.55 ± 0.57	
Left hepatic duct (LHD)	2.85 ± 1.16	1.18 - 7.04
Male	2.79 ± 1.04	
Female	2.89 ± 1.24	
Common hepatic duct (CHD)	2.19 ± 1.01	1.00 - 5.10
Male	2.25 ± 1.18	
Female	2.14 ± 0.89	
Common bile duct (CBD)	7.21 ± 2.19	3.19 - 12.89
Male	7.75 ± 1.94	
Female	6.81 ± 2.28	
Cystic duct (CD)	2.86 ± 1.68	0.40 - 8.38
Male	3.52 ± 1.83	
Female	2.39 ± 1.41	
Pancreatic duct (PD)	8.63 ± 1.85	5.14 - 11.89
Male	8.99 ± 1.77	
Female	8.49 ± 1.88	

	Mean angle and standard deviation (°)	Range (°)
Cysticohepatic angle (CHA)	51.46 ± 14.59	17.90 - 94.20
Male	54.56 ± 16.19	
Female	49.25 ± 13.01	
Hepatic bifurcation angle (HBA)	72.34 ± 21.26	25.00 - 123.80
Male	66.48 ± 19.03	
Female	76.53 ± 21.89	

We found no relation between CBD length and gender (P = 0.1); however, there was positive correlation between CBD length and age as its length increases with age (P = 0.04).

Abnormal anatomical variations were noticed in 36 patients (30%). Short CD (> 1 cm) was found in 24 patients (20%). Left CD insertion was in six patients (5%). Two of the patients (1.7%) have their CD inserted in the RHD. Duct of Luschka, which is abnormal small duct connecting the liver directly with the gall bladder, was seen in four patients (3.33%). Accessory hepatic duct was found in also four patients (3.33%).

The observed anatomical variations of EHBT and PD, their frequencies and percentages are shown in Table 2.

**Table 2:** Anatomical Variations of EHBT and PD

	Frequency (n)	Percentage (%)
Cystic duct (CD)		
Long CD (< 4 cm)	18	15.00
Normal CD length (1 - 4 cm)	78	65.00
Short CD (< 1 cm)	24	20.00
High insertion	88	73.30
Mid insertion	30	25.00
Low insertion	2	1.70
Right insertion	114	95.00
Left insertion	6	5.00
CD inserted in RHD	2	1.70
Duct of Luschka	4	3.33
Cystico-hepatic duct angle		
Acute	118	98.30
Wide	2	1.70
Hepatic Bifurcation angle		
Acute	96	80.00
Wide	24	20.00
Hepatic duct		
Accessory hepatic duct	4	3.33
Pancreatic duct		
Major papillae insertion	77	64.00
Minor papillae insertion	43	36.00

Out of the 120 patients who underwent ERCP and/or MRCP, stones were positive in 86 patients (71.7%). Sixty-eight of the patients (56.7%) underwent cholecystectomy. Biliary tract injuries were reported in 18 patients (15%). Biliary tract injuries were higher in abnormal anatomy (P = 0.04), but there was no relation between abnormal anatomy and stones formation (P = 0.07).

## Discussion

EHBT anatomy is of great importance to the surgeon since EHBT is one of the common sites of anatomical variations besides being one of the most common sites for surgical procedures. Incidence of EHBT abnormal anatomy varies; it was reported to be as high as 47% [5]. In a study by Kullman et al (1996), EHBT anatomical variations were found in 19% of their patients [6]. Another study by Hasan et al (2013) reported incidence of 15.2% [7], while it was observed to be lower by Philippo et al (2008) (8.8%) and Cochoeira et al (2012) (7.3%) [8, 9]. In this study, we found abnormal anatomy in 30% of the cases which is higher compared to other studies.

Proper identification of EHBT anatomy and its possible abnormalities would allow surgeons to perform safe operation with no or minimal injuries. It is important to the surgeons to be aware of the most common abnormalities. The most EHBT abnormality observed in our study was short CD which was found in 20% of the patients. This finding coincided with studies by Talpur et al (2010) and Khan et al (2012) where short CD was reported as the most common abnormality. However, a study by Kullman et al and another by Devi et al (2013) reported that accessory hepatic ducts are the most common EHBT abnormalities [10]. In this study, accessory hepatic ducts were observed in only 3.33%.

The second most common abnormality we found was left CD insertion which was in 5%. Bicaj et al (2013) studied the variations of CD insertions and found left CD insertion in 20.95% which is much higher compared to our finding [11]. Another study by Talpur et al reported that incidence of left CD insertion is 10-17%. Less common abnormalities in our study are CD inserted into RHD (1.7%) and duct of Luschka (3.33%). In a study by Filippo et al, CD inserted into RHD in 2.7 %. Nearly, the same finding was reported by Cachoeira et al. Lower percentages of duct of Luschka were reported by Talpur et al and Khan et al (0.67% and 1.7%) respectively.

Biliary tract injury was reported in 15% in our study, which is higher compared to other studies. This may be attributed to the higher incidence of abnormal anatomy in the region. We found an association between abnormal anatomy and biliary tract injury (P = 0.04). The same association was reported by Hasan et al with biliary tract injury incidence of 6%. Lower incidence was reported by Kallman et al and Krahenbuhl (2001) (0.5% and 0.3%) respectively [11, 12]. Hence, it is recommended that surgeons properly identify the EHBT anatomy intraoperatively in order to avoid injuries.

To our knowledge, there are no studies in the literature that looked at the association between abnormal anatomy of EHBT and stone formation. According to this study, the association seems to be statistically insignificant (P = 0.07).

### Conclusion

Abnormal anatomy of EHBT was found to be 30%. The most common abnormality was short CD followed by left CD insertion. Surgeons should be aware of these common abnormalities in this region, hence avoiding injury to the biliary tract during surgery. The abnormal anatomy was associated with high incidence of biliary tract injury but had no relation to biliary stone formation.

## References

[1] Lamah M, Dickson GH (1999). Congenital anatomical abnormalities of the extrahepatic biliary duct: a personal audit. Surg Radiol Anat.

[2] Talpur KA, Laghari AA, Yousfani SA, Malik AM, Memon AI, Khan SA (2010). Anatomical variations and congenital anomalies of extra hepatic biliary system encountered during laparoscopic cholecystectomy. J Pak Med Assoc.

[3] Khan A, Paracha S, Shah Z, Tahir M, Wahab M (2012). Anatomical Variations of Cystic Duct Encountered During Open Cholecystectomy. KMUJ.

[4] Saad N, Darcy M (2008). Iatrogenic bile duct injury during laparoscopic cholecystectomy. Tech Vasc Interv Radiol.

[5] Dundaraddy R, Mahesh G (2012). Study of Variations in the Extrahepatic Biliary System. Biomirror Journal.

[6] Kullman E, Borch K, Lindstrom E, Svanvik J, Anderberg B (1996). Value of routine intraoperative cholangiography in detecting aberrant bile ducts and bile duct injuries during laparoscopic cholecystectomy. Br J Surg.

[7] Hasan MM, Reza E, Khan MR, Laila SZ, Rahman F, Mamun MH (2013). Anatomical and congenital anomalies of extra hepatic biliary system encountered during cholecystectomy. Mymensingh Med J.

[8] De Filippo M, Calabrese M, Quinto S, Rastelli A, Bertellini A, Martora R, Sverzellati N (2008). Congenital anomalies and variations of the bile and pancreatic ducts: magnetic resonance cholangiopancreatography findings, epidemiology and clinical significance. Radiol Med.

[9] Cachoeira E, Rivas A, Gabrielli C (2012). Anatomic Variations of Extrahepatic Bile Duct and Evaluation of the Length of Ducts Composing the Cystohepatic Triangle. Int.J.Morphol.

[10] Devi T, Krishna P (2013). The Study of Variations of Extra Hepatic Biliary Apparatus. IOSR-JDMS.

[11] Bicaj B, Qamirani S, Blakaj F, Zejnullahu V, Hamza A (2013). Variations of Cystic Duct (CD)-Common Hepatic Duct (CHD) Junction Determned by ERCP. Med Arh.

[12] Krahenbuhl L, Sclabas G, Wente MN, Schafer M, Schlumpf R, Buchler MW (2001). Incidence, risk factors, and prevention of biliary tract injuries during laparoscopic cholecystectomy in Switzerland. World J Surg.

